# Annotations

**Published:** 1907-12-07

**Authors:** 


### ANNOTATIONS.

### Royal College of Surgeons Annual Meeting.

The annual meetings of the Fellows and Members of the Royal College of Surgeons of England have a decidedly uniform or even monotonous quality. A small but energetic section of the Members attends on these occasions and presents an unvarying demand for representation on the governing Council of the College. A resolution in this direction is passed unanimously, the President undertakes to present it to the Council, and all is quiet until the following year brings a repetition of the performance. On the present occasion, however, a new element was introduced as a result of the representations which had been made on behalf of the Members to the Privy Council. This appeal produced from the Lord President a suggestion that the Council should formally express to him its reasons for resisting the claims advanced by the Members of the College. In this way the Council has been forced to depart from its attitude of superiority and silence, and to attempt to justify its negative decisions. It now appears that the fear is lest the representation of members should be followed by "socio-political" discussions, in which "the present class of councillors would decline to take part." The argument is a familiar one in connection with representative institutions of all kinds, but whether it can be regarded as effective is a different question. The members are not, we think, always very happy in their prominent advocates, but, in our judgment, they have an undeniable case and will sooner or later be successful. They may secure some leverage out of the decision of the Council to submit to them the question whether or not women should be admitted to the College examinations. But they will hardly vindicate their character for liberality and good judgment if they endorse the argument of one of their number, that, as women have access to many other examining boards, they ought to be excluded from that of the College of Surgeons.

### Pharmacopœial Revision.

The process of time has now brought well within view the issue of another edition of the British Pharmacopœia—a responsible duty which is placed by law upon the shoulders of the General Medical Council. At the recent meeting of the Council a report was received from the committee specially concerned with the Pharmacopœia, and from this it is possible to gather some hints as to the principles upon which the work is conducted. The last edition of the official volume was issued in 1898, and has since the date of its appearance been the subject of continuous criticism. This criticism has, in its turn, to be examined and valued, while at the same time the appearance of new remedies and modern pharmaceutical developments must be borne in mind. It may thus be fairly said that no sooner has one edition of the Pharmacopœia made its appearance than preparations have to be commenced for the issue of its successor. Much interest in the recent report attaches to the statement that the committee has been engaged in the collection of statistics as to the frequency with which particular medicines, both official and non-official, are actually prescribed in the United Kingdom. For this purpose upwards of 43,000 prescriptions dispensed in various parts of the country have been examined and analysed. The inference from such a proceeding is that the committee continues to recognise that the Pharmacopœia exists mainly to secure on the part of dispensers a uniform interpretation of the terms used in physicians' prescriptions. The public interest demands that such terms shall be authoritatively defined; and this the Pharmacopœia by its legal authority is able to secure. It is, therefore, the duty of such a volume to admit to its pages all drugs which are shown by experience to have a reasonably extensive area of employment. There are some authorities who would make the Pharmacopœia a list of substances possessing a secure pharmacological definition. But the General Medical Council has never adopted this view, and it is satisfactory to find that the test of public service is still to be the guide in this matter. The Pharmacopœia, in short, exists, not to announce an official view of what drugs are, and what are not, of therapeutic value, but to ensure that uniformity in the strength and quality of medicines which is essential to the safety and interest of the community.

### Reform in Medical Nomenclature.

America has given us a vast number of new medical names and synonyms. Indeed, it seems that where a disease has two or more names, the one well known and generally accepted, the others obsolete, obscure, or less well naturalised, the American chooses one of the latter, and adds to the confusion of the English reader by dressing it up in Americanised spelling. Some of the names our brethren on the other side of the Atlantic have invented for certain operations are veritable masterpieces of philological ingenuity. But unfortunately the English language does not share with the Greek or the German the characteristic of felicitously lending itself to involved "compound-formation" of words, and some of these new coinages are not only confusing, but distinctly awkward. In an address before the Johns Hopkins Medical Society, Dr. Rotch pleads for the simplification of medical indices. Many of the titles given to diseases, he remarks, are meaningless and incorrect, mere relics, in fact, of past ignorance, clogging the wheels of progress, conveying false ideas, and leading to confusion in classification and discussion. There is a great deal of truth in this indictment. The tendency to "visualise the whole disease in its name" is both unfortunate and wrong, and it is probable that as our knowledge grows we shall have to modify, if not completely to change, our nomenclature. The principle of tacking to a disease the name of its first describer—Todd, Parkinson, Friedreich, Flagani, and others—has at least this point in its favour, that, while not introducing a title that attempts a finality impossible to realise at present, it associates the disease, or symptom group to which the name is applied, with an historical event in the progress of medicine, and therefore to the student at least it serves as a useful mnemonic.

